# Source of cigarettes among youth smokers in Malaysia: Findings from the tobacco and e-cigarette survey among Malaysian school adolescents (TECMA)

**DOI:** 10.18332/tid/96297

**Published:** 2018-11-05

**Authors:** Kuang Hock Lim, Chien Huey Teh, Pei Pei Heng, Sayan Pan, Miaw Yn Ling, Muhammad Fadhli Mohd Yusoff, Sumarni Mohd Ghazali, Chee Cheong Kee, Rafiza Shaharudin, Hui Li Lim

**Affiliations:** 1 Institute for Medical Research, Kuala Lumpur, Malaysia; 2 Jabatan Kesihatan Negeri Perlis, Perlis, Malaysia; 3 Institute of Public Health, Kuala Lumpur, Malaysia; 4 Hospital Sultan Haji Ahmad Shah, Temerloh, Malaysia

**Keywords:** source of cigarettes, Malaysian youth, frequent smokers, TECMA

## Abstract

**INTRODUCTION:**

Understanding how and where youth obtain tobacco products are major factors in the development of suitable intervention programs to reduce youth smoking. This study aimed to determine the source of cigarettes and the associated factors among Malaysian school adolescent smokers.

**METHODS:**

Our sample consisted of 1348 youth aged 10–17 years who were current smokers (having smoked at least once in the last 30 days). The source of cigarettes (commercial, over-the-counter purchases; or social, borrowing or obtaining from someone else) was the dependent variable, and multivariable logistic regression was employed to determine its association with independent variables (i.e. sociodemographics, smoking behavior, and knowledge of laws prohibiting sales of cigarettes to youth).

**RESULTS:**

Over half (54.3%) of current smokers obtained cigarettes from commercial sources, with a proportion nearly two times higher (84.2% vs 43.7%) among frequent smokers (i.e. those smoking more than 20 days per month) compared to less-frequent smokers, and among young males (56.5% vs 32.0%) compared young females. Multivariable logistic regression indicated that in urban areas, young females (AOR=12.5, 95% CI: 1.38–99.8) frequent smokers (AOR=4.41, 95% CI: 2.05–9.46), and those studying in lower (AOR=3.76, 95% CI: 1.41–10.02) and upper secondary (AOR=4.74, 95% CI: 1.72–13.06) school students were more likely to obtain cigarettes from a commercial source. On the other hand, in rural areas, only frequent smokers were more likely to get their cigarettes from commercial sources, whilst other variables were not significant.

**CONCLUSIONS:**

The proportion of youth smokers who obtained cigarettes from commercial sources appeared to be high, suggesting that law enforcement and health promotion activities should be enhanced to reduce the rate of smoking among Malaysian youth.

## INTRODUCTION

Tobacco use is the most preventable cause of morbidity and mortality in Malaysia. About 20000 deaths are reported annually^1^ and smoking-related diseases have been identified as major contributors to disability-adjusted life years (DALYs) among the Malaysian population^2^. The Malaysian government has enacted comprehensive measures involving various agencies and non-governmental organizations (NGOs) to address health issues related to smoking, by promoting healthy lifestyle campaigns, engaging in advocacy, introducing smoke-free areas, raising the prices of tobacco products by changing the tax structure of cigarettes from kilograms to sticks, and implementing community intervention programs such as KOSPEN (*Komuniti Sihat, Pembina Negara* — Healthy community, developed the nation)^3^. Such initiatives^4^ are intended to decrease the prevalence of smoking among Malaysian adults to 15% by the year 2025.

Various studies have shown that smoking behavior is learned and begins during adolescence^5,6^. A similar phenomenon has been reported in Malaysia, where over 80% of adult smokers began smoking before the age of 21 years^1^. According to the theory of human development, most adolescents start smoking during adolescence because of abstract thinking, common during that phase, which contributes to the occurrence of ‘Formal Operations’ and ‘Personal Fable’^7^, when adolescents feel that they are unique and omnipotent. In addition, they might also feel that people around them are eagerly watching or listening to them; the feeling of the presence of an imaginary audience and a sense of invincibility^8^ might drive adolescents (younger than 18 years old) to engage in risky health behaviors, such as smoking, to attract the attention of their peers. Furthermore, the earlier a young person begins smoking, the more likely he or she will continue to do so because of the addictive effects of tobacco products^9^. This pattern decreases a person’s likelihood to quit smoking^10^ and increases the risk of smoking-related illness, such as cancer^11^. Therefore, reducing smoking initiation among adolescents aims at lowering the prevalence of smoking among Malaysian adults that will ultimately reduce smoking-related morbidity and mortality among Malaysians.

Measures to prevent adolescents from accessing tobacco products have included educational efforts imploring them to choose wisely, and make rational, mature decisions regarding their behaviour, including smoking^12^. In addition, making cigarettes less available to youth has indirectly encouraged them to smoke less frequently and less willing to share their cigarettes^13^. These measures have been identified as some of the best strategies to reduce smoking initiation, as shown by various studies, including those related to reducing smoking initiation among adolescents^14^, along with decreasing the progression of experimental smokers to frequent smokers^15^.

The World Health Organization (WHO) developed the Framework Convention on Tobacco Control, which urged all member countries to take pro-active measures to reduce the supply of tobacco to adolescent smokers to discourage them from smoking^16^. Through the Ministry of Health, the Malaysian government has implemented similar measures, including prohibitions on the ownership, use, and purchase of tobacco products by individuals under 18 years, as well as a ban on the sale of tobacco products to adolescents through tobacco control regulations enacted in 2004. Additionally, the country has banned retail sales (i.e. selling tobacco sticks) and set a minimum price for cigarettes^17^.

Research has revealed that adolescents generally obtain tobacco products from social sources^18^. In addition, epidemiology studies^18,19^ have shown that older teenagers, males and heavy smokers^18,19^ are more likely to buy cigarettes from a commercial store. The opposite is true for young female smokers. Similarly, infrequent smokers of both genders are more likely to obtain tobacco from peers and other adults^20^.

In Malaysia, limited research has explored the procurement of tobacco products among adolescent smokers; most studies have focused on the prevalence of and factors related to smoking among adolescents. Only three small-scale studies have investigated tobacco procurement by adolescents over the last two decades^21-23^. Those studies pertained to specific localities among upper Secondary school students only^21-23^; thus, their findings cannot be generalized outside these regions and groups. Furthermore, the sociodemographics of respondents in those localities (i.e. Kota Tinggi, and Petaling) were different to those of Malaysian adolescents, in term of age, and ethnicity.

The adaptation of findings from Western countries concerning the procurement of tobacco products may not be applicable to Malaysia, due to social and cultural differences, the dynamics of smoking among adolescents, and the level of enforcement against cigarette procurement. Therefore, current information on how Malaysian adolescent smokers obtain cigarettes is of paramount importance, given the paucity of data available. Such findings will assist in discovering loopholes in the existing law and enforcement activities in order to curb adolescent access to cigarettes. The present study illustrates the sources of cigarettes, and the associated factors, for school adolescent smokers in Malaysia.

## METHODS

We conducted a nationwide study entitled ‘Tobacco and E-Cigarette survey among Malaysian Adolescents’ (TECMA), in 2016, to obtain the latest data on tobacco and e-cigarette use among Malaysian school adolescents. A cross-sectional study design with multiple-stage stratified cluster sampling was used to select a representative sample size of Malaysian school adolescents, 10–19 years old, based on the latest sampling frame of upper Primary and Secondary school enrolment provided by the Ministry of Education (DOE) of Malaysia. The first strata consisted of 15 States in Malaysia, and the second included their subdivision into urban and rural areas. The primary sampling unit was either the Secondary or Primary schools available in each State, selected based on proportion to size sampling approach, followed by class selection (i.e. secondary sampling unit) from each chosen school. All students from the selected classes were invited to participate in the study. In total, 138 schools were selected (82 urban and 56 rural). The sample size was determined using an estimated prevalence of 3% e-cigarette users among adolescents in Korea^24^ at a 95% confidence interval (CI) with a design effect to account for any cluster effects among students in the classes. We also set a tolerable error of 1.5% and expected a non-response rate of 20%. Based on these parameters, 13980 respondents were required for the study. A total of 13162 adolescents participated in the study, yielding a response rate of 88.7% (13162/14832).

### Measures

An active informed consent approach was used to obtain permission from the parents/guardians of the selected respondents. Specifically, informed consent forms were distributed to parents/guardians of the selected respondents to explain the study objectives. Respondent participation was voluntary, all participants were assured of anonymity, and data were used for research purposes only. Respondents’ parents/guardians were asked to return the informed consent form if they would allow their child to participate. Only selected respondents who returned the informed consent form could participate in the study. Data collection was carried out in a designated area identified by school administrators during the school day. To avoid the Hawthorne effect (tendency of research subjects to act atypically as a result of their awareness of being studied), school teachers and staff were asked not to be present while students completed the questionnaire.

Members of the research team provided a detailed overview of the study, prior to questionnaire completion. The briefing included information on the study objectives, contents of the questionnaire, and assurance that all participation was voluntary; students could skip any item(s) on the survey. Research team members assisted any respondents who did not understand certain items on the questionnaire. In addition to consent from students’ parents/guardians, selected respondents were asked to sign an additional consent form if they agreed to participate in the study.

A validated questionnaire was used in the TECMA study. The questionnaire was adapted from the Global Youth Tobacco Survey (GYTS), the Global School Health Survey (GSHS), and input from content experts. All items in the GYTS and GSHS questionnaires were translated into Bahasa Malaysia by a panel of language and content experts, and was back-translated by another group of language and content experts to ensure the content and meaning of the items remained intact. The questionnaire was pre-tested among chosen students in selected Primary and Secondary schools in Kuala Lumpur to ensure that assessment items suited the local sociocultural context and to establish face validity. Minor corrections and modifications were applied to the questionnaire based on student pre-test feedback.

The questionnaire included the sections: 1) *sociodemographics*, i.e. age, gender, standard/form of study (upper Primary, lower Secondary, upper Secondary), ethnicity, daily school pocket money; 2) *tobacco use*, e.g. current use status, age of initiation; 3) *e-cigarette use*, i.e. current status, number of quitting attempts; and 4) *shisha use*, i.e. current status, age of initiation. The study protocol was approved by Malaysia’s Ministry of Education, Ministry of Health, and State Education Department; ethical approval was granted by the Medical Research and Ethics Committee of Malaysia’s Ministry of Health.

Only respondents under 18 years old who claimed they had ever tried or experimented with cigarette smoking, even one or two puffs, and smoked at least one day during the last 30 days (i.e. current smokers) were included in the analysis; students aged 18 years and older were excluded because they were of legal age to purchase tobacco. The dependent variable was ‘source of cigarettes’. The dependent variable was measured using the item: ‘The last time you smoked cigarettes during the past 30 days (one month), how did you get them?’. Respondents could select more than one response option, including: ‘I bought them in a supermarket, grocery store or roadside stall’, ‘shared the cost of cigarettes with my friends’, ‘I paid someone to buy for me’, ‘I borrowed them from someone else’, ‘I stole them’, ‘I got them from my family’, and ‘I got them from someone else’. Respondents who answered, ‘I bought them in a supermarket, grocery store or roadside stall’ and other combinations were categorized as having bought cigarettes from ‘commercial and social sources’, whereas those who selected a combination of answers other than ‘I bought them in a supermarket, grocery store or roadside stall’ were categorized as having obtained cigarettes from a ‘social source’.

Independent variables in the study were gender, form of study (i.e. upper Primary, lower Secondary, upper Secondary), ethnicity (i.e. Malay, Chinese, Indian, Bumiputra Sabah, Bumiputra Sarawak), locality (i.e. urban/rural), type of smoker (i.e. frequent smoker: smoked 20 days or more during the last 30 days; infrequent smoker: smoked less than 20 days during the last 30 days). Awareness of tobacco regulations regarding cigarette procurement was measured using three items: 1) ‘Smoking under [the] age of 18 is an offence under the law’; 2) ‘Buying cigarettes under the age of 18 is not an offence under the law’; and 3) ‘Having cigarettes under the age [of] 18 is an offence under the law’ with response options of ‘yes’, ‘no’, or ‘don’t know’. A correct answer was given 1 point, and a wrong answer zero. The final score on knowledge about laws prohibiting adolescent smoking and possession of cigarettes ranged from 0–3 points, with a higher score indicating better knowledge of the law.

### Data analysis

Data were cleaned and weighted based on the study design and non-response rate prior to analysis. Descriptive statistics were used to illustrate respondent demographic characteristics. Chi-squared analysis was used to determine the association between ‘source of cigarettes’, including all categorical independent variables. Independent t-tests were used to compare the different mean scores on legal knowledge between current smokers who obtained cigarettes from ‘social source only’ and ‘social and commercial sources’. Multivariable logistic regression (MLR) was conducted to determine factors associated with cigarette sources.

Independent variables were included in the model to determine the effect of each on the dependent variable (i.e. source of cigarettes), after adjusting for the influence of other confounding factors. All two-way interactions between independent variables in the final model were also examined. The interaction analysis revealed a significant interaction between locality/residence areas and gender. Therefore, an MLR for urban and rural was run to determine the factors associated with the source of cigarettes based on locality. All statistical analyses were run at a 95% CI using SPSS software version 20.

## RESULTS

Overall, 13.5% of school adolescents were current smokers, with a significantly higher proportion among Bumiputra Sabah adolescents (16.1%) followed by Malay (13.8%). The study showed that the ratio of male to female current smokers was more than 10:1, and the prevalence of smoking among school adolescents in rural areas was almost twice as high as their urban counterparts (7.8%, 95% CI: 7.0–8.7 for urban; 14.9%, 95% CI: 13.5–16.4 for rural). Approximately 21.7% of current smokers reported smoking frequently (Table 1).

**Table 1:** Sociodemographics and frequency of smoking among Malaysian school adolescent current smokers, 2016 (n=1348 )

*Variable*	*Estimated population*	*Sample*	*%*	*95% CI*	*p*
**Overall**	409386	1348	13.5	13.1–13.9	
**Gender**					
Male	374177	1248	21.1	19.6–22.6	<0.001
Female	35209	100	2.0	1.5–2.8	
Residential*					
Urban	125359	661	7.8	7.0–8.7	<0.001
Rural	283990	687	14.9	13.5–16.4	
**Level of schooling**					
Upper Primary	76218	289	5.8	4.9–6.7	<0.001
Lower Secondary	201498	601	14.6	13.9–16.3	
Upper Secondary	131669	508	16.4	14.6–18.4	
**Ethnicity**					
Malay	320003	1104	13.8	12.6–15.0	<0.001
Chinese	13948	32	3.1	2.2–4.4	
Indian	8485	31	4.1	2.7–6.3	
Bumiputra Sabah	31772	77	16.1	12.5–20.5	
Bumiputra Sarawak	20278	45	10.5	7.6–14.2	
Others	14898	39	10.4	7.1–14.7	
**Frequent smoker**					
Yes	88726	272	21.7	18.5–25.2	<0.001
No	320660	1076	83.7	74.8–81.2	

* Urban Area – a gazetted administrative area with adjoining built-up areas of 10000 people or more. Rural area – a gazetted administrative area of less than 10000 people is defined as a rural area, frequent smoker: current smokers who smoked 20 days or more during the last 30 days.

Table 2 shows that over half (54.3%) of current smokers obtained cigarettes from a store; the proportion was significantly higher among young males (56.5%, 95% CI: 51.7–61.1) than young females (32.0%, 95% CI: 17.5–50.9), and for current smokers from upper Secondary school (59.6%, 95% CI: 52.4–66.4) compared to those from upper Primary school (32.7%, 95% CI: 24.3–42.3). In addition, the proportion of frequent smokers who bought cigarettes from a shop was nearly twice as high compared to infrequent smokers (84.2%, 95% CI: 77.0–89.4; and 43.7%, 95% CI 38.3–49.3, respectively; p<0.001). Multivariate analysis (Table 3) by locality revealed that, in urban settings, female current smokers (AOR=12.5, 95% CI: 1.38–99.8), those from lower Secondary school (AOR=3.76, 95% CI: 1.41–10.02) and upper Secondary school (AOR=4.41, 95 % CI: 1.72–13.06), with upper Primary school as reference, and frequent smokers (AOR=4.41, 95% CI: 2.25–9.46), were more likely to buy cigarettes from shops compared with young males, upper Primary school students, and infrequent smokers. However, no similar trend was observed among smokers from rural settings; only frequent smokers were more likely to buy cigarettes from a shop (AOR=6.19, 95% CI: 2.41–15.88). No significant association was found between ethnicity and knowledge of smoking regulations with buying cigarettes in either urban or rural areas.

**Table 2:** Type of cigarette source among school adolescent smokers by sociodemographics, Malaysia, 2016 (n=1014 )

*Variable*	*Social source only*				Commercial source*			
	*Estimated population*	*Sample*	*%*	*95% CI*	*Estimated population*	*Sample*	*%*	*95% CI*	*p*
**Overall**	145732	483	45.7	41.1–50.4	173284	531	54.3	49.6–58.9	
**Gender**									
Male	126463	441	43.5	38.9–48.3	1642350	505	56.5	51.7–61.1	0.012
Female	19369	42	68.0	49.1–82.5	9048	26	32.0	17.5–50.9	
**Residential**									
Urban	43485	237	48.2	42.2–54.1	46786	259	51.8	45.9–57.8	0.424
Rural	102246	246	44.7	38.7–50.8	126497	272	55.3	49.2–61.3	
**Level of schooling**									
Upper Primary	33774	108	67.3	57.7–75.7	16373	46	32.7	24.3–42.3	<0.001
Lower Secondary	64996	215	21.5	35.4–50.2	87575	211	57.4	49.8–64.6	
Upper Secondary	46961	160	40.4	33.6–47.6	69335	274	59.6	52.4–66.4	
**Ethnicity**									
Malay	111601	398	44.7	39.4–50.2	137906	435	55.3	49.8–60.6	0.365
Chinese	3788	15	40.5	23.5–60.1	5576	23	59.5	39.9–76.5	
Indian	2373	8	43.6	19.5–71.1	3070	9	56.4	28.9–80.5	
Bumiputra Sabah	12844	29	47.9	33.2–62.9	13990	31	52.1	37.1–66.8	
Bumiputra Sarawak	7651	15	44.3	27.7–62.3	9622	22	53.7	37.7–72.3	
Others	7473	18	70.6	49.4–85.5	3119	11	29.1	14.5–50.6	
**Frequent smoker**									
Yes	13215	50	15.8	10.6–23.0	70270	207	84.2	77.0–89.4	<0.001
No	132516	433	56.3	50.7–61.7	103014	324	43.7	38.3–49.3	

* Commercial source – cigarettes from supermarket, grocery store or roadside stall. Social source – cigarettes other than from commercial source.

**Table 3:** Association between sociodemographics and frequency of smoking cigarettes from commercial sources by locality (urban/rural) among adolescent current smokers in Malaysia, 2016 (n=708 )

*Variable*	*Urban (n=354 )*		*Rural (n=354 )*	
	*AOR*	*95% CI*	*AOR*	*95% CI*
**Gender**				
Male	1			
Female	12.5	1.38–99.8	0.60	0.14–2.56
**Level of schooling**				
Upper Primary	1		1	
Lower Secondary	3.76	1.41–10.02	1.70	0.71–4.07
Upper Secondary	4.74	1.72–13.06	1.39	0.54–3.61
**Ethnicity**				
Malay	1		1	
Chinese	2.26	0.65–7.79	–	
Indian	1.43	0.27–7.54	–	
Bumiputra Sabah	2.44	0.21–28.1	0.51	0.19–1.33
Bumiputra Sarawak	0.96	0.26–3.56	0.70	0.24–2.05
Others	0.67	0.18–2.51	0.33	0.01–1.19
**Frequent smoker**				
Yes	4.41	2.05–9.46	6.19	2.41–15.88
No	1			
**Knowledge of smoking regulation**	0.96	0.65–1.40	1.39	0.89–2.19

## DISCUSSION

Our study found that over half of current smokers (54.3%) obtained cigarettes from commercial sources, contradicting studies in developed countries reporting that adolescent smokers obtain cigarettes from social sources^18,25^. The proportion of adolescents who obtained cigarettes from commercial sources was 3.5- to 5.5-fold higher compared with youth in Minnesota, in 2003 and 2011 (16.3% and 9.7%, respectively). Findings from a longitudinal study by Robinson et al.^26^ revealed that 16% of 4416 youth smokers obtained their cigarettes from a commercial source. The prevalence of cigarettes obtained from commercial sources in the present study was much lower than that reported by Lim et al.^22,23^ among Secondary school students in Kota Tinggi, Johor^22^, and Petaling Jaya, Selangor^23^, as it was by Zulkifli and Rogayah^21^ among youth smokers in Kelantan. However, the prevalence is worrisome given that legislation prohibiting the sale of tobacco products to individuals under 18 years old has been enforced for over a decade. We postulated that the higher proportion in our study might be due to the wide availability of cigarettes in Malaysia, where such products can be sold by any business, such as sundry shops, restaurants, and service stations without restrictions. No licensing mechanisms are in place to control cigarette sales, and a lack of manpower to uphold such policies may also be problematic.

Currently, the power to enforce tobacco product sales is only granted to the Medical Officer of Health, through the environmental officers/assistants EHOs (EHOs/AEHOs) in the district health department, who are heavily involved in prevention and control activities of communicable and non-communicable diseases in their respective districts; therefore, they cannot focus fully on enforcing tobacco control activities. Detailed studies should be carried out in this aspect to identify the actual factors contributing to our findings.

Male school adolescents from urban areas were significantly less likely to purchase cigarettes compared with female smokers. This finding was quite surprising given the higher prevalence of smoking among young men in Malaysia, and our finding is not congruent with that of Kaestle et al.^18^, Lim et al.^22,23^ and Minaker et al.^27^, all of whom reported that men were more likely to obtain cigarettes from commercial sources. This finding of the present study could be due to several factors, such as the tendency of adolescent male smokers to befriend male peers who smoke, thus promoting social connectivity and togetherness^28^. In this case, young men habitually obtain cigarettes and smoke together with groups of friends on their way to and from school, at the train or bus station, or when socializing; as such, smoking becomes a social signifier within peer groups where they consume and exchange cigarettes together for group inclusion^3^. By comparison, female smokers, who constitute a small proportion of smokers and are therefore less likely to have social support to obtain cigarettes, access cigarettes independently and over the counter. Girls of similar age may appear physically older due to developmental differences; thus, sales people may be more mistrustful of boys and subsequently attribute negative motives to boys but not to girls of an equivalent age^29^. Consistent findings from other studies suggest that underage girls may have less trouble purchasing cigarettes than boys^30^. Additionally, social bonding among urban communities might not be as strong as in rural regions due to the environment and longer working hours, resulting in less time to mingle in neighborhoods. Urban residents are also more self-centered; such communities typically disregard or are oblivious to underage girls buying cigarettes from a store^31^.

Together with previous studies^18,25^, our study demonstrated that age is significantly associated with increased use of tobacco and commercial access but decreased social access. As the age of youth increases, physical and psychological development is not advanced; hence, adolescents struggle against dependency. This phenomenon is well described in the adolescent development literature^7,8^. Upper Secondary students appear to be more mature physically and are more fearless, courageous, heroic, and apt to explore risky situations. This age group may begin to admire adult smokers; according to social cognitive theory, humans learn behaviors by observing others^32^. Imitation is more likely when observers admire those they are observing. The developmental perspective posits that adolescent behaviors are motivated by the desire to assume adult characteristics^8^. Therefore, higher-grade students may be more confident when making over-the-counter cigarette purchases. This trend aligns with our findings that upper Secondary students are more likely to obtain cigarettes from commercial sources, followed by lower Secondary and upper Primary students. Furthermore, older youth may be better able to afford cigarettes via commercial routes, as teenagers from upper Secondary school may earn money through a part-time job and thus be able to afford over-the-counter purchases. Tyas and Pederson^33^ indeed reported that a higher number of adolescent smokers bought their own cigarettes, had a part-time job, and earned personal income. However, similar patterns were not observed in rural areas; no significant association emerged between gender, level of schooling and ethnicity, with obtaining cigarettes via commercial sources. We postulated that social bonding among residents in rural areas could explain this finding, with traditional values (e.g. positive attitudes and values of friendliness, helpfulness, hospitality, and neighborly behavior) prevalent among rural residents. Families are closely bonded to one another and are aware of and have acknowledge on the issues happening around them. The value of community or society precedes that of an individual. Some adolescent smokers may perceive society and their parents/guardians as being against smoking^5,23^; therefore, adolescents may be less likely to become involved in activities (e.g. direct cigarette purchases) that could tarnish their image with authority figures.

Frequent smokers in our study were more likely to purchase their own cigarettes in rural and urban localities after adjusting for the effects of confounding factors. Similar findings have been reported in other research^25,27^. Regular smokers typically identify common stores that offer easy access to cigarettes, such as grocery stores and convenience stores. As frequent buyers, they may be familiar with certain premises where they can procure cigarettes illegally. Forster et al.^20^ and Minaker et al.^27^ similarly discovered that daily smokers make most tobacco purchases to win friends by offering cigarettes to peers. Frequent smokers may also be more likely to buy cigarettes in stores due to tobacco addiction. Addiction is a dynamic process that typically begins with occasional smoking and later progresses to frequent. Frequent smokers find quitting difficult when they are already addicted to smoking. More advanced stages of smoking are greatly associated with purchasing their last cigarette^25,27^. Most youth began to purchase their own cigarettes once dependence sets in from increased levels of cigarette consumption^27^. The frequent smokers might also patronize the same premises to buy cigarettes, thus cultivating a close relationship with the shop owner and purchasing cigarettes more easily from that store.

This study also revealed that knowledge of smoking regulations was not associated with buying cigarettes in rural or urban settings. With the proliferation of laws prohibiting underage tobacco sales, we expected that access to cigarettes among underage youth would be stricter. We hypothesize that inadequate regulation enforcement could contribute to this finding. The EHOs/AEHOs enforce Control of Tobacco Product Regulation (CTPR) in Malaysia^17^. Nevertheless, manpower is extremely limited in conducting compliance checks at all tobacco retailers. These public health inspectors can barely oversee all premises. Moreover, charges and penalties only apply when shop owners or salespeople are directly caught selling tobacco products to minors^14^. Lack of enforcement and burden of proof may cause adolescent smokers to believe that the probability of being caught purchasing tobacco is low; thus, they may not worry about breaking a law that prohibits them from buying cigarettes. However, more studies should be carried out to verify the absence of a significant association between knowledge of relevant tobacco laws and cigarette sources.

This research has several limitations. First, we did not include an appraisal of knowledge and attitudes of retailers regarding legislation prohibiting tobacco sales to persons under 18 years of age. Second, cigarette sources were self-reported; respondents may have under- or over-reported this information.

## CONCLUSIONS

This study showed that adolescents can easily access commercial sources of cigarettes in Malaysia. Current selling mechanisms in the country allow any store to sell tobacco; therefore, store owners can participate freely in the tobacco products business without restrictions. Laws concerning the sale and distribution of cigarettes could potentially affect adolescent tobacco use and decrease youth access to tobacco. To accomplish this, licensing of tobacco outlets may prove helpful; specifically, establishing a licensing system for cigarette sales could be an effective regulatory tool. Under this system, all stores that sell tobacco would be required to obtain a special license to do so. This measure would allow the government to better monitor youth access to tobacco, marketing exposure, retailer density, and retailer location. Under such a licensing system, licensees would be required to verify a cigarette buyer’s age through proof of identity and age, to avoid violating regulations. In addition, health promotion activities should be intensified to raise retailer awareness about existing laws prohibiting cigarette sales to adolescents.
